# Yield strength and misfit volumes of NiCoCr and implications for short-range-order

**DOI:** 10.1038/s41467-020-16083-1

**Published:** 2020-05-19

**Authors:** Binglun Yin, Shuhei Yoshida, Nobuhiro Tsuji, W. A. Curtin

**Affiliations:** 1 Laboratory for Multiscale Mechanics Modeling (LAMMM) and National Centre for Computational Design and Discovery of Novel Materials (NCCR MARVEL), École Polytechnique Fédérale de Lausanne, 1015 Lausanne, Switzerland; 2 Department of Materials Science and Engineering, Kyoto University, Kyoto, 606-8501 Japan; 3 Elements Strategy Initiative for Structural Materials (ESISM), Kyoto University, Kyoto, 606-8501 Japan

**Keywords:** Mechanical properties, Metals and alloys, Atomistic models

## Abstract

The face-centered cubic medium-entropy alloy NiCoCr has received considerable attention for its good mechanical properties, uncertain stacking fault energy, etc, some of which have been attributed to chemical short-range order (SRO). Here, we examine the yield strength and misfit volumes of NiCoCr to determine whether SRO has measurably influenced mechanical properties. Polycrystalline strengths show no systematic trend with different processing conditions. Measured misfit volumes in NiCoCr are consistent with those in random binaries. Yield strength prediction of a random NiCoCr alloy matches well with experiments. Finally, we show that standard spin-polarized density functional theory (DFT) calculations of misfit volumes are not accurate for NiCoCr. This implies that DFT may be inaccurate for other subtle structural quantities such as atom-atom bond distance so that caution is required in drawing conclusions about NiCoCr based on DFT. These findings all lead to the conclusion that, under typical processing conditions, SRO in NiCoCr is either negligible or has no systematic measurable effect on strength.

## Abstract (web summary)

Chemical short-range order (SRO) NiCoCr has been proposed to account for its positive stacking fault energy and good mechanical properties. Here, a combination of theory and experiment shows that SRO is of negligible importance in NiCoCr processed by standard methods.

## Introduction

The multicomponent, non-dilute high/medium-entropy alloys (HEAs/MEAs) are a new class of structural metal alloys. HEAs/MEAs are under intense study due to intriguing combinations of properties such as high strength and high fracture toughness for certain alloys^1–5^. The MEA fcc NiCoCr was shown to have the highest yield strength among the Cr–Mn–Fe–Co–Ni family of equi-composition fcc alloys^6^ and a phenomenal fracture toughness at low temperature^4^. The stable stacking fault energy (SFE) of NiCoCr was measured to be 22 ± 4 mJ m^−2^^7^ whereas first-principles density-functional theory (DFT) studies on the random alloy showed a negative SFE at low temperatures, possibly becoming positive only around 300 K^8^. Chemical short-range order (SRO) in NiCoCr has been suggested as the reason for the positive SFE, based on DFT^9^. SRO was also deduced from EXAFS experiments but the interpretation relied on DFT^10^. A new interatomic potential for the Ni–Co–Cr system fitted to extensive DFT suggested the SRO should be evident even at high annealing temperatures (e.g., 1650 K) and with a significant enhancement of the tensile yield strength as compared with the random alloy^11^. While the interatomic potential is only a model NiCoCr alloy, implications were made for real NiCoCr experiments.

With the above high attention given, and implications made^9–11^, about SRO in NiCoCr processed and reported to date, in this paper we address the issue of whether SRO has any demonstrable effects on measurable mechanical properties in NiCoCr prepared by standard processing routes. Statistical mechanics dictates that SRO is always non-zero if specific atom–atom interactions are non-zero, and so it is not possible to prove that SRO does not exist. The issue is then whether the SRO is (i) negligible (undetectably low), (ii) present at measurable levels but having negligible effects on mechanical properties, or (iii) present and with distinct effects on properties as claimed in recent literature.

Here, literature experiments on tensile strength are first examined, and show no indication of strength variations with processing conditions. Long-time lower-temperature annealing experiments are then shown to have no effect on the hardness of NiCoCr as compared with the initial sample annealed at high temperature. Precise measurements of the lattice constants of NiCoCr and surrounding compositions allow for an accurate determination of the Ni, Co, and Cr misfit/apparent volumes, which generally agree with those found for random Ni_2_Co and Ni_2_Cr binary alloys. The measured misfit volumes and single-crystal elastic constants then enable application of a theory to predict the initial yield strength of a random alloy and good agreement with experimental values is obtained. With the misfit volumes in NiCoCr carefully measured here for the first time, we then show that first-principles DFT calculations are not accurate for the misfit volumes in NiCoCr, which has implications for the previous analysis of SRO from EXAFS experiments^10^ and for the development of interatomic potentials based solely on DFT inputs^11^. Based on all of these findings, we can conclude that, in the NiCoCr alloys fabricated and tested to date, SRO is either negligible or exists but does not have any important effect on yield strength and misfit volumes.

## Results

### Strength of polycrystalline in NiCoCr versus processing

Different levels of SRO would be expected for different annealing temperatures. Therefore, we start with an examination of yield strength and hardness of polycrystalline in NiCoCr over various processing conditions (a range of annealing temperatures, primarily) to explore any signatures of SRO.

**Fig. 1:**
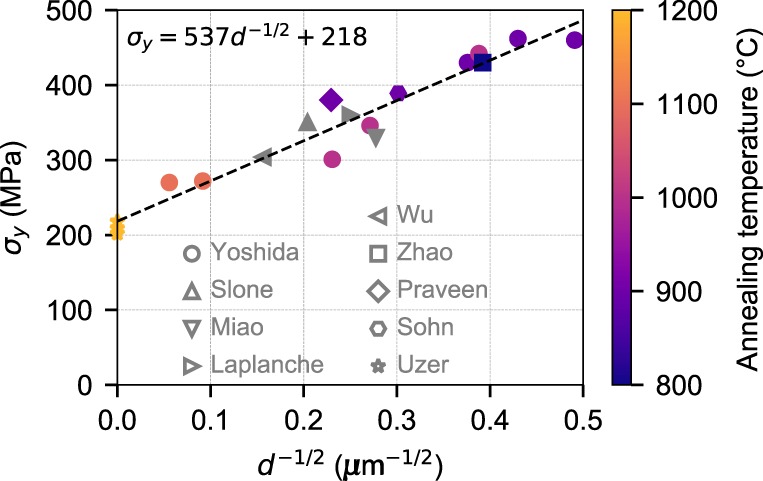
Aggregated literature data on yield strength versus inverse square-root of grain size in NiCoCr (Hall–Petch scaling). All samples are single-phase fcc with grain size *d* ≥ 4 μm (excluding twin boundaries) and tested in tension at room temperature and strain rate 0.5–1 × 10^−3^ s^−1^. Samples indicated in color were water-quenched after the final heat treatment, with the color indicating the heat treatment temperature. Samples in gray were air-cooled after heat treatment. A best-fit linear regression to all of the data is shown, and differences in heat treatment and/or cooling method do not give rise to measurable differences in strength. The strength obtained by extrapolation to infinite grain size agrees well with the recent single-crystal experiments (data of Uzer et al.^18^).

We have carefully curated literature data on the uniaxial tensile yield strength *σ*_*y*_ of NiCoCr versus grain size *d*^6,7,12–18^, as shown in Fig. 1. Most samples are homogenized at a fairly high temperature (1100–1200 °C), so this feature is not indicated. For water-quenched samples, the recrystallization annealing temperature used in fabricating each tensile sample is indicated by the color map. Air-cooled samples are indicated in gray since the annealing temperature is not likely relevant. If these different treatments induce SRO, and if SRO affects the yield strength, then all the experimental results summarized in Fig. 1 should not follow one single Hall–Petch (HP) scaling relationship. However, all the aggregated data in Fig. 1 do follow just one HP relationship. The scatter in the data is comparable to that found for fixed processing (data of Yoshida et al.^12^), indicating no notable trends of strengthening with annealing temperature and/or cooling method. The extrapolated strength of 218 MPa at infinite grain size matches well with recent single-crystal data (211 ± 9 MPa, obtained from measured critical resolved shear stress of 69 ± 3 MPa^18^ multiplied by the Taylor factor of 3.06 to obtain the uniaxial tensile strength of an untextured fcc polycrystal). Within the uncertainty of the strength data, the literature yield strength of NiCoCr does not show any significant consequences of SRO.

The experiments associated with Fig. 1 were usually performed with short annealing time (≤1 h) and high temperatures. Here, we show that longer-time annealing at lower temperatures has negligible effect on the hardness of NiCoCr. We fabricate NiCoCr samples by casting, cold rolling to 50% thickness reduction, and homogenizing (recrystallization annealing at the same time) at 1100 °C for 24 h. Using this starting material, two samples are extracted and annealed further for 384 h at 700 and 600 °C, respectively. Water quenching is performed after every heat treatment. There are no changes in grain size after the long-time annealing relative to the starting material (~50 μm); this is consistent with our extensive data on grain size versus annealing time and temperature that indicates expected changes in grain size to be <1 μm. From diffusion data on NiCoCr^19^, solute diffusion distances of several nm at 600 °C, and much larger at 700 °C, are expected for this long-time annealing, sufficient for the development of non-negligible SRO if thermodynamically significant at these temperatures. The Vickers hardness of the three samples, all with textures close to random, are measured to be 163 ± 3.4 HV (starting material), 164 ± 2.9 HV (700 °C), and 163 ± 3.2 HV (600 °C), respectively, i.e., essentially identical. If SRO is to arise, different levels of SRO are expected in these long-time annealed samples as compared with the initial sample. The identical hardness results here (typically corresponding to a plastic flow stress at ~7–10% plastic strain) indicate that any SRO (either negligible or measurable) under these conditions has no measurable effect on mechanical performance.

SRO might be induced by annealing at lower temperatures (e.g., 500 °C), as suggested by literature data on Ni_2_Cr^20^ and Ni_2_Cr-1 at.% Fe^21^, and by thermodynamic studies on NiCoCr and other Cr-containing Cantor-like alloys^22,23^. Recent work reported SRO and increased strength and hardness in NiCoCr, but on samples that were furnace-cooled, rather than water-quenched or air-cooled^24^. The observed SRO is thus likely formed during cooling in the range *T* < 600 °C, which would be fully consistent with existing literature and the present work. However, such processing conditions are not typical, and speculations for the role of SRO^9–11^ have been related to alloys processed at conditions corresponding to those for Fig. 1.

### Misfit volumes in NiCoCr

The misfit volume of an element in a given alloy reflects the average local atomic environment around the element. We thus precisely measure the misfit volumes for Ni, Co, and Cr in NiCoCr and compare against random binary data of Ni–Co and Ni–Cr to seek any significant differences that might reflect the existence of SRO.

The misfit volume of the type-*n* solute (*n* = 1…*N*) in an *N*-component alloy can be expressed in terms of derivatives of alloy atomic volume with respect to composition. The alloy atomic volume can be expressed by the compositions of any *N* − 1 of the components since $\mathop {\sum}\nolimits_n {c_n = 1}$, so $V_{{\mathrm{alloy}}} = V_{{\mathrm{alloy}}}(c_1,c_2,...,c_{N - 1})$ and $\partial V_{{\mathrm{alloy}}}{\mathrm{/}}\partial c_N = 0$. The misfit volume of any type-*n* atom Δ*V*_*n*_ can then be calculated as

1 $$\Delta V_n = \frac{{\partial V_{{\mathrm{alloy}}}}}{{\partial c_n}} - \mathop {\sum}\limits_{m = 1}^N {c_m} \frac{{\partial V_{{\mathrm{alloy}}}}}{{\partial c_m}},$$

which can be derived from related expressions given in ref. ^25^.

**Fig. 2:**
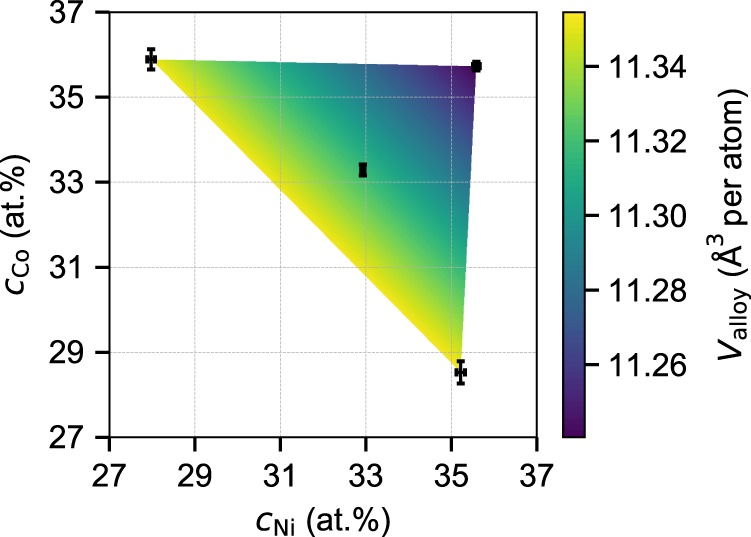
Measured composition and atomic volume of Ni–Co–Cr samples at four compositions around the equi-composition alloy. The actual compositions with the uncertainties are shown by the black symbols. The color map indicates the alloy atomic volume computed by a linear interpolation of the measured volumes.

To obtain the derivatives required in Eq. (1), we fabricate four single-phase fcc Ni–Co–Cr alloy samples with compositions around the equi-composition NiCoCr by casting, cold rolling to 50% thickness reduction, and homogenizing at 1100 °C for 50 h, followed by cold rolling to 80% thickness reduction and annealing at 900 °C for 15 min. Precise measurements of the actual compositions (uncertainty < ±0.26 at.%) and lattice constants (uncertainty < ±0.001 Å) are performed (see “Methods”). The associated alloy atomic volumes *V*_alloy_ (*V*_alloy_ = *a*^3^/4) versus Ni and Co composition are shown in Fig. 2 with uncertainties indicated. Linear regression using the atomic volumes *V*_alloy_ of these four alloys leads to the alloy atomic volume versus composition as $V_{{\mathrm{alloy}}} = 12.2997 - 1.48309c_{{\mathrm{Ni}}} - 1.48630c_{{\mathrm{Co}}}$ with *R*^2^ = 0.9999. This analysis immediately gives the precise lattice constant of NiCoCr at the exact composition (3.5632 Å), which is well within the literature range of 3.5595–3.5696 Å^7,10,23,26,27^. Then, most importantly, we determine the misfit volumes of Ni, Co, and Cr in NiCoCr as −0.493 Å^3^, −0.497 Å^3^, and +0.990 Å^3^, respectively.

**Fig. 3:**
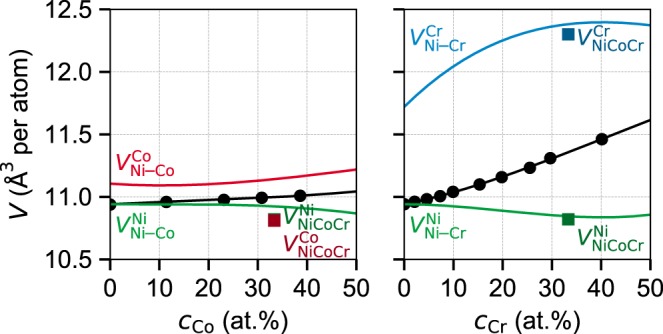
Alloy atomic volumes for Ni–Co and Ni–Cr binary alloys versus Co and Cr composition (black symbols), respectively, and apparent volumes $V_{{\mathrm{alloy}}}^{{\mathrm{solute}} - n}$ for Ni, Co, and Cr versus composition (colored lines). Also shown are the apparent volumes for Ni, Co, and Cr in NiCoCr as measured in Fig. 2 and shown at the Ni_2_Co and Ni_2_Cr compositions (colored symbols).

For simplicity in comparisons here, we consider the apparent atomic volume for each element in the alloy defined as $V_{{\mathrm{alloy}}}^{{\mathrm{solute}} - n} = V_{{\mathrm{alloy}}} + \Delta V_n$. The apparent volumes in NiCoCr are compared with those in the binary Ni_2_Co and Ni_2_Cr random alloys (annealed at high temperatures, 800 and 1200 °C, respectively, and water-quenched)^28,29^. For Ni–Co and Ni–Cr alloys, we first take the literature *V*_alloy_(*c*_X_) (X = Co or Cr) over a range of concentrations^28,29^ and fit the data to cubic polynomials, as shown in Fig. 3. We then compute the misfit volumes using Eq. (1) and then obtain the apparent volumes as shown in Fig. 3. The apparent volume of Ni in NiCoCr as measured here is very close to those found for Ni in both Ni_2_Co and Ni_2_Cr. The apparent volume for Cr in NiCoCr as measured here is also very close to that found for Ni_2_Cr. The apparent volume for Co in NiCoCr is slightly lower by ~0.3 Å^3^ than that found in Ni_2_Co; this is not unexpected due to the magnetic interactions between Co and Cr and the strong dependence of Co atomic volume on the Co magnetic moment (as established in DFT studies, including ours as discussed below). The local environments around each element in NiCoCr show no clear difference from the random binary alloys, and so provide no evidence for the existence of SRO.

### Solute strengthening in NiCoCr: theory versus experiment

With the experimental misfit volumes and two recent literature values for the room temperature single-crystal elastic constants^30,31^, we can predict the initial yield strength of NiCoCr within the framework of an existing solute strengthening theory for random alloys^32,33^, which has previously shown quantitative success for a number of HEAs^25,32–36^. We can then compare the random alloy strength prediction against experiments.

The theory computes the zero-temperature yield shear stress *τ*_*y*0_ and the energy barrier Δ*E*_*b*_ for thermally activated flow as

2 $$\tau _{y0} = A_\tau \left[ {\frac{\Gamma }{{b^2}}} \right]^{ - \frac{1}{3}}\left[ {\mu ^V\frac{{1 + \nu ^V}}{{1 - \nu ^V}}} \right]^{\frac{4}{3}}\delta ^{\frac{4}{3}},\\ \Delta E_b = A_E\left[ {\frac{\Gamma }{{b^2}}} \right]^{\frac{1}{3}}b^3\left[ {\mu ^V\frac{{1 + \nu ^V}}{{1 - \nu ^V}}} \right]^{\frac{2}{3}}\delta ^{\frac{2}{3}}.$$

Here, *b* is the Burgers vector; Γ = *αμ*_110/111_*b*^2^ is the dislocation line tension; *α* = 0.125 is the edge dislocation line tension parameter, accurate for a number of fcc metals; *μ*_110/111_ is the shear modulus for fcc slip on the {111} plane in the 〈110〉 direction; *μ*^*V*^ and *ν*^*V*^ are the Voigt average of shear modulus and Poisson’s ratio; $\delta = \sqrt {\mathop {\sum}\nolimits_n c_n\Delta V_n^2} /(3V_{{\mathrm{alloy}}})$ is the well-known *δ*-parameter describing the collective effect of the individual solute misfit volumes Δ*V*_*n*_; the prefactors $A_\tau = 0.04865\left[ {1 - (A - 1){\mathrm{/}}40} \right]$ and $A_E = 2.5785\left[ {1 - (A - 1){\mathrm{/}}80} \right]$ are associated with a typical fcc dislocation core structure consisting of two well-separated partial dislocations plus a small correction for elastic anisotropy^37^ via the Zener anisotropy *A* = 2*C*_44_/(*C*_11_ − *C*_12_). At temperature *T* and strain rate $\dot \epsilon$, standard thermal activation theory then leads to the predicted shear yield strength as

3 $$\tau _y(T,\dot \epsilon ) = \tau _{y0}\left[ {1 - \left( {\frac{{kT}}{{\Delta E_b}}\ln{ \frac{{\dot \epsilon _0}}{{\dot \epsilon }}} } \right)^{\frac{2}{3}}} \right],$$

where $\dot \epsilon _0$ is a reference strain rate estimated to be 10^4^ s^−1^^34^. The uniaxial tensile yield strength *σ*_*y*_ of an isotropic polycrystal is obtained by multiplying *τ*_*y*_ by the Taylor factor 3.06. Application of the theory thus requires only the alloy lattice constant, elastic constants, and the elemental misfit volumes in the alloy at the composition of interest.

From the experimental misfit volumes and lattice parameter for NiCoCr, we compute the *δ* parameter as *δ* = 0.02063. From the elastic constants, we obtain the Zener anisotropy *A* = 3.0 and *μ*_110/111_ = 78 GPa. Application of the theory at *T* = 300 K and strain rate 5 × 10^−4^ s^−1^ (corresponding to the single-crystal experiments) leads to a predicted strength of 195 MPa. This value is in good agreement with both the measured single-crystal and HP-extrapolated strengths, as summarized in Table 1. The strength of NiCoCr is well-captured by the theory without considering any SRO. While the theory has underlying approximations and is certainly not exact, this agreement again suggests that any SRO that might be present has a negligible effect on yield strength relative to a random alloy.

**Table 1:** Material parameters and the resulting yield strength prediction at *T* = 300 K and strain rate 5 × 10^−4^ s^−1^ for NiCoCr and 10^−4^ s^−1^ for Ni_2_Cr.

	NiCoCr		Ni_2_Cr	
	Exp RT	DFT	Exp RT	DFT
*a*_0_ (Å)	3.563	3.528	3.568 (derived in Fig. 3)	3.546
*δ* (%)	2.063	(1.109)	2.128 (derived in Fig. 3)	(1.550)
*C*_11_ (GPa)	252^30,31^	262	–	230
*C*_12_ (GPa)	158^30,31^	163	–	140
*C*_44_ (GPa)	142^30,31^	139	–	126
*A*	3.0	2.8	–	2.8
*μ*^*V*^ (GPa)	104	103	–	94
*ν*^*V*^	0.268	0.276	–	0.267
*μ*_110/111_ (GPa)	78	79	–	72
*μ*^*H*^ (GPa)	91	92	–	83
*μ*^poly^ (GPa)	87^6^	–	80^20^	–
*σ*_*y*_ (MPa) (predicted)	195		163	
*σ*_*y*_ (MPa) (measured)	211 (from single-crystal experiments^18^)		~150^20^	
	218 (from Hall–Petch scaling derived in Fig. 1)			

DFT *δ*-parameters are in parentheses indicating that these results are unreliable.

To further support our conclusions based on the theory for alloy strength, we apply the strength theory to Ni_2_Cr. The Ni–Cr random binary alloys up to 40 at.% Cr were studied by Clement et al.^20^ at large grain size (300 μm)^38^ and explicitly processed to avoid SRO^20^. A polycrystal isotropic shear modulus of *μ*^poly^ = 80 GPa is the only elastic modulus information reported^20^. So, we use first-principles DFT to compute the elastic constants of Ni_2_Cr and NiCoCr as shown in Table 1; the agreement with experiment for NiCoCr provides confidence in the results for Ni_2_Cr. Also, the DFT-computed Voigt–Reuss–Hill shear modulus *μ*^*H*^ is in close agreement with the measured isotropic polycrystalline modulus *μ*^poly^ for both NiCoCr and Ni_2_Cr. Then, using the experimental misfit volumes and lattice constant (see Fig. 3) and the DFT-computed elastic constants, we predict the yield strength of Ni_2_Cr to be 163 MPa at the experimental strain rate of 10^−4^ s^−1^. The experimental strength is ~150 MPa, as obtained by interpolating quoted results of 22 and 40 at.% Cr^20^. Within the uncertainty of the inputs and theory, agreement between theory and experiment on random Ni_2_Cr is good. The misfit parameter in the Ni_2_Cr alloy without SRO is quite similar to NiCoCr, and the lower strength arises mainly due to lower elastic moduli. These similarities support our conclusion that any SRO in NiCoCr studied to date has no discernable effect on yield strength.

### Deviations of first-principles DFT from experiments

It has been very common to apply standard spin-polarized DFT to compute properties and understand atomic structures and local atomic distortions in HEAs^8,9,17,39–41^. It is well-established that DFT predicts lattice constants and elastic constants can differ moderately from experiments. Table 1 shows that DFT predictions of elastic constants in random NiCoCr and Ni_2_Cr are in reasonable agreement with experiments, suggesting DFT may be generally applicable to NiCoCr. DFT may also provide reasonable results for the energetic driving forces that could cause SRO in NiCoCr^9,41^. However, with accurately measured solute misfit volumes, we can demonstrate that, unfortunately, DFT provides very poor results for the misfit volumes in NiCoCr. This makes DFT possibly unsuitable for quantitative use in drawing conclusions about local atomic environments.

**Fig. 4:**
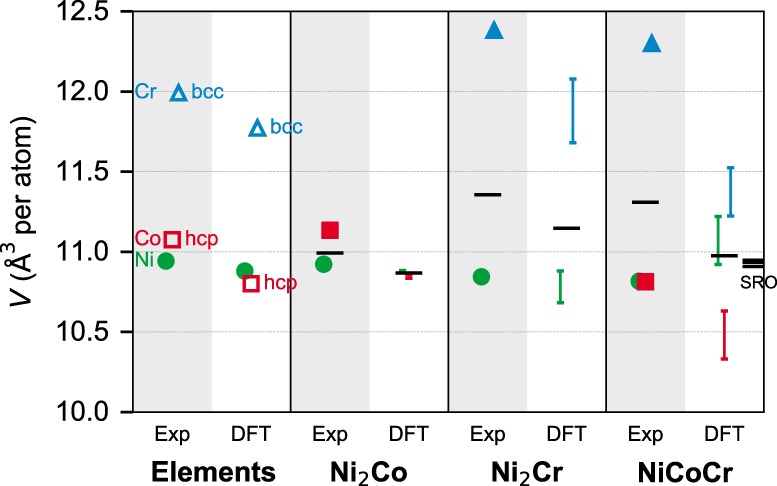
Apparent volumes of Ni (green), Co (red), and Cr (blue) in various fcc alloys from room temperature experiments and DFT. The error bar in DFT data indicates the 95% confidence interval in the linear regression in the misfit volume calculation. Black bars are the alloy atomic volumes. All the results are from fcc structure unless indicated and shown as hollow symbols.

Our Vienna ab initio simulation package (VASP)-special quasi-random structure (SQS)^42,43^ results (see “Methods”) for alloy atomic volumes and solute apparent volumes are shown in Fig. 4. Our computed alloy atomic volumes are consistent with literature DFT studies^8,9,17,44^. The uncertainties in apparent volumes in Fig. 4 are the 95% confidence intervals for each linear regression in the calculation of misfit volumes, arising due to the varying atomic and/or magnetic random configurations of the samples^25^. The uncertainty of the alloy atomic volumes *V*_alloy_ is rather small (<±0.01 Å^3^) and are therefore neglected. We first examine the elements and the binary alloys. The elemental experimental volumes for fcc Ni, hcp Co, and bcc Cr, and their DFT-computed volumes reveal that the atomic volume of Ni at *T* = 0 K is well reproduced by DFT (ferromagnetic)^45^ and so the difference between experiments at 300 K and DFT of bulk Ni in Fig. 4 is likely due to thermal expansion. In contrast, the DFT volumes of hcp Co (ferromagnetic) and bcc Cr (antiferromagnetic) are noticeably smaller than experiments. For Ni_2_Co, the DFT alloy atomic volume is underpredicted relative to experiments, but consistent with the error in the elemental DFT volumes. The experiments show a small but clear increase in the alloy atomic volume by adding Co into Ni (see Fig. 3), and so it is the DFT apparent volume of Co that is too small in Ni_2_Co. For Ni_2_Cr, the DFT alloy atomic volume is again lower than experiment, but the decrease in DFT apparent volume of Ni relative to the elemental value is consistent with the experiments. More importantly, the DFT apparent volume for Cr increases relative to the elemental value but much less than found in experiments. The DFT misfit volumes of Ni and Cr in Ni_2_Cr are thus both rather smaller than experiments. In spite of the differences in volumes, the net DFT magnetic moments of ~1 μ_B_ per atom for Ni_2_Co and ~0.05 μ_B_ per atom for Ni_2_Cr are consistent with the experiments^46^.

For NiCoCr, the deviations between DFT and experiments are more glaring. Experiments show that the alloy atomic volume and apparent volumes of Ni and Cr are nearly the same as in Ni_2_Cr. However, the DFT alloy atomic volume decreases notably relative to Ni_2_Cr. The DFT apparent volume of Ni increases while experiments show little change, and the DFT apparent volumes of both Co and Cr drop considerably relative to Ni_2_Co and Ni_2_Cr, respectively. The overall DFT predictions for NiCoCr are thus quantitatively very poor. The significant underpredictions of the misfit volumes would lead to significant underpredictions of the *δ* parameter (see Table 1) and hence of the yield strength. The deviations in DFT for NiCoCr compared with experiments might be thought to arise due to SRO in the experiments. However, the DFT-computed alloy atomic volumes for NiCoCr with three different levels of SRO^9^, as shown in Fig. 4, show a further but small decrease in alloy atomic volume as compared with the random alloy. So, it is highly unlikely that SRO is the reason for the deviations—rather, it is the DFT that is in error. These large errors for NiCoCr indicate that DFT is not sufficiently reliable for understanding local structural features of NiCoCr, such as the misfit volumes and the atom–atom bond distances that are measured in EXAFS experiments^10^.

The origins of the volume decrease of Co in NiCoCr appear to stem from magnetic interactions between Co and Cr and the strong magnetism-volume correlation in Co atoms. The atomic volume of Co itself strongly depends on the magnetic moment. DFT calculations of fcc Co show a decrease in atomic volume of ~0.6 Å^3^ from the ferromagnetic state with magnetic moment 1.66 μ_B_ to the nonmagnetic state. In NiCoCr, literature DFT results^40,41^ already showed that the magnetic moments of Co atoms are significantly reduced due to the Co–Cr interaction, fluctuating around 0.9 μ_B_ rather than the elemental Co value (1.66 μ_B_). We have also been able to correlate SQS-to-SQS atomic volume variations with variations in the average Co moment, with smaller Co moments showing smaller volumes. This rationalizes why the apparent Co volume in NiCoCr is significantly smaller than that in Co and Ni_2_Co, in both experiments and DFT. This also rationalizes why the DFT NiCoCr volume shrinks further in the presence of SRO, which increases the number of Co–Cr neighbors^9^. Finally, the net moment in NiCoCr as computed in DFT is ~0.3 μ_B_ per atom^40,41^ whereas real NiCoCr is paramagnetic above 4 K^47^.

In addition to VASP-SQS, we have also used the exact muffin-tin orbitals (EMTO)-coherent potential approximation (CPA)^48^ method (see “Methods”) to compute the misfit volumes in Ni_2_Cr and NiCoCr. Initializing the alloy with disordered local moments, both Ni_2_Cr and NiCoCr relax to essentially nonmagnetic states, with atomic moments being smaller than 0.01 μ_*B*_. In these two nonmagnetic cases, the apparent volumes of Cr are close (~11.74 Å^3^) and also close to nonmagnetic VASP-SQS values (~11.63 Å^3^, not shown in Fig. 4). All of these values remain, however, far below the experimental value (12.30 Å^3^). For NiCoCr, ferromagnetic initialization leads to a slightly lower-energy state compared with the nonmagnetic state, with a net moment of 0.26 μ_B_ per atom (see also ref. ^49^). In this magnetic state, the apparent volume of Cr (11.24 Å^3^) shows a much larger decrease than in the nonmagnetic state, similar to the VASP-SQS results shown in Fig. 4. Overall, the apparent volumes computed from EMTO-CPA are consistent with the VASP-SQS results. But both methods fail to predict accurate misfit volumes in NiCoCr, with or without spin polarization.

## Discussion

Our analyses and results for strengths and misfit volumes of NiCoCr lead us to conclude that, in the existing NiCoCr experiments and processing methods to date, SRO is negligible to the extent that it has no measurable influence on the individual misfit volumes or alloy strength and hardness. This conclusion does not rule out possible SRO formation under some processing conditions (e.g., annealing at ~500 °C). As noted earlier, a recent study does report evidence of SRO and consequent effects in NiCoCr—a strengthening of ~50 MPa^24^. The processing in that study involved furnace cooling after annealing at 1000 °C for 120 h, and the SRO likely develops in these materials during the slow furnace cooling in the regime *T* < 600 °C. This would then be fully consistent with literature development of SRO in this temperature range and, moreover, with our finding of no change in hardness after even longer annealing at both 600 and 700 °C.

We further show that the high strength reported for NiCoCr, relative to the Cantor alloy and its subsystems, need not be attributed to SRO but rather can be understood based solely on the misfit volumes and elasticity of the random alloy. We also show that standard spin-polarized DFT does not accurately predict the misfit volumes of NiCoCr, indicating inaccuracy in subtle structural results like atom–atom bond distance. This shows that care must be taken in using DFT results as a basis for (i) interpreting EXAFS experiments^10^, (ii) drawing conclusions about various structural properties of NiCoCr, or (iii) creating interatomic potentials^11^.

Finally, while the creation of SRO in NiCoCr may remain of scientific interest, the pursuit of high-performance alloys in various classes of HEAs/MEAs is likely better achieved by exploring new compositions across wider classes of alloying elements. For example, a strength of ~400 MPa has recently been reported for NiCoV^17^, nearly double that of (random) NiCoCr. The strength of NiCoV is also well-predicted by the random alloy solute strengthening theory^35^. Thus, the modest strengthening (~50 MPa) that might be achieved by inducing SRO in NiCoCr under particular processing conditions may be of limited practical value. More generally, it is difficult to measure and control SRO, and there are no theories to predict the strengthening caused by SRO, especially in complex alloys. The successes to date of the strength theory for random alloys thus points to use of the random alloy theory for guiding alloy design, followed by careful experiments on the promising alloy compositions identified by the theory.

## Methods

### Experiments

The misfit volumes for Ni, Co, and Cr in NiCoCr are measured as follows.

Ingots of Ni–Co–Cr alloys with compositions around the NiCoCr equi-composition are fabricated by vacuum arc-melting of constituent elements with a purity of more than 99.9 wt.% and casting in a water-cooled copper mold under protective Ar atmosphere. The ingots are then cold rolled with a reduction of 50% in thickness (to accelerate diffusion later by introducing lattice defects) and homogenized at 1100 °C for 50 h to remove segregation of elements completely. The resulting materials are in nearly random texture, with grain size of ~100 μm. Afterward, the materials are cold rolled again with a reduction of 80% in thickness and subsequently annealed at 900 °C for 15 min. After this processing, fully-recrystallized, nearly random textured microstructures are obtained, which are in homogeneous fcc single-phase with mean grain sizes of several μm. Water quenching is conducted after every heat treatment.

The specimens for the proceeding experiments are cut from the annealed materials by a wire cutting machine (Brother, HS-70A) into a dimension of 30 (in length) × 8 (in width) × 0.5 mm (in thickness). Afterward, the specimens are mechanically polished by 1000–4000 grid-sized fine sandpapers and then 1–3 μm diamond pastes to achieve mirror-like surfaces.

Wavelength dispersive X-ray (WDX) spectroscopy is employed to analyze the chemical composition of the materials. WDX measurements are performed in a scanning electron microscope (SEM, Hitachi, S-3500H) equipped with a LiF WDX spectrometer (MICROSPEC, WDX-3PC). The working distance is set at 15 mm for the measurements. The acceleration voltage in SEM is maintained at 25 kV. The current of electron beam is precisely measured by using Faraday cup. The calibration of the system is performed using a standard pure Fe sample. Pure Ni, Co, and Cr are used as reference samples for the measurements. The chemical composition of randomly selected 10 points on the surface (normal direction plane for rolling sheets) are analyzed in each specimen.

The lattice constants are determined by X-ray diffraction (XRD) technique. The XRD measurements are performed at room temperature (*T* = 25 °C) in a synchrotron facility, beamline No. 46XU in Super Photon Ring-8 GeV (SPring-8) in Hyogo, Japan. The energy of incident X-ray beam is set to be 30 keV (wavelength of 0.0413 nm) by using an automated double-mirror monochromator, and the spot size of the beam is 0.3 (in length) × 0.5 mm (in width). Each specimen is set on a computer-controlled four-axis goniometer so that the position of the specimen is adjusted exactly as same as designated camera length of the system, and the surface is perfectly parallel to the incident X-ray beam. The transmission-diffracted X-ray beam is detected by a one-dimensional semiconductor detector, and the data acquisition time (exposure time) is set to be 60 s to achieve better signal–noise ratio. The calibration of the system is done using a standard CeO_2_ sample.

### Computations

Collinear spin-polarized DFT computations as implemented in the VASP^42^ are performed using a supercell method with the PBE functional^50^. The valence states for the elements are Ni (4*s*, 3*d*), Co (4*s*, 3*d*), and Cr (3*p*, 4*s*, 3*d*), employed with PAW pseudopotentials^51^. The valence-electron eigenstates are expanded using a plane wave basis set with a cutoff energy 550 eV. In reciprocal space, a Γ-centered Monkhorst–Pack^52^ *k*-mesh is used with line density consistent across all geometries. The interval between the neighboring *k*-points along each reciprocal lattice vector **b**_*j*_ is 0.02 Å^−1^ (in VASP, **a**_*i*_**·b**_*j*_ = *δ*_*ij*_). This *k*-mesh density leads to, for example, 14 × 14 × 14 for Ni in the fcc cubic unit cell. A first-order Methfessel–Paxton method^53^ with smearing parameter 0.2 eV is used. Ionic forces are relaxed to <1 meV Å^−1^, corresponding to a stress tolerance of ~0.01 GPa in VASP. The magnetic moments are initialized in a ferromagnetic configuration with 1 μ_B_ per atom, and the directions and magnitudes adjust during the electronic relaxation. The resulting magnetic moment distributions are consistent with literature DFT studies^40,41^.

SQSs^43^ with 108 atoms are generated by the ATAT code^54^ at central compositions A_2_B (for Ni_2_Co and Ni_2_Cr) and ABC (for NiCoCr), as well as the surrounding compositions. This supercell size has been shown to be sufficient to achieve accurate misfit volumes in a number of other systems^2,25^. Misfit volumes are computed using a procedure identical to that used experimentally, but executed on 15–30 SQSs at various compositions. Details can be found in ref. ^25^.

The CPA is implemented using the EMTO method^48^. We again use the PBE functional^50^ in both the self-consistent electronic density calculation and the total energy calculation.

## Data Availability

The data that support the findings of this study are available from the corresponding authors upon reasonable request.
